# A microRNA-1280/JAG2 network comprises a novel biological target in high-risk medulloblastoma

**DOI:** 10.18632/oncotarget.2779

**Published:** 2014-12-17

**Authors:** Fengfei Wang, Marc Remke, Kruttika Bhat, Eric T. Wong, Shuang Zhou, Vijay Ramaswamy, Adrian Dubuc, Ekokobe Fonkem, Saeed Salem, Hongbing Zhang, Tze-chen Hsieh, Stephen T. O'Rourke, Lizi Wu, David W. Li, Cynthia Hawkins, Isaac S. Kohane, Joseph M. Wu, Min Wu, Michael D. Taylor, Erxi Wu

**Affiliations:** ^1^ Department of Pharmaceutical Sciences, North Dakota State University, Fargo, ND 58105, USA; ^2^ Arthur and Sonia Labatt Brain Tumor Research Centre, Program in Developmental and Stem Cell Biology, Hospital for Sick Children, University of Toronto, Toronto, ON M5G 1X8, Canada; ^3^ Brain Tumor Center & Neuro-Oncology Unit, Department of Neurology, Beth Israel Deaconess Medical Center, Harvard Medical School, Boston, MA 02115, USA; ^4^ Scott & White Neuroscience Institute, Texas A & M Health Science Center, Temple, TX 76508, USA; ^5^ Department of Computer Sciences, North Dakota State University, Fargo, ND 58105, USA; ^6^ Department of Physiology, State Key Laboratory of Medical Molecular Biology, Institute of Basic Medical Sciences, Chinese Academy of Medical Sciences and Peking Union Medical College, Beijing 100073, China; ^7^ Department of Biochemistry and Molecular Biology, New York Medical College, Valhalla, NY 10595, USA; ^8^ Department of Molecular Genetics and Microbiology, Shands Cancer Center, University of Florida, Gainesville, FL 32610, USA; ^9^ Department of Ophthalmology & Visual Sciences, College of Medicine, University of Nebraska Medical Center, Omaha, NE 68198, USA; ^10^ Division of Pathology, Hospital for Sick Children, Toronto, ON M5G 1X8, Canada; ^11^ Informatics Program, Children's Hospital Boston, Harvard Medical School, Boston 02115, MA, USA; ^12^ Department of Biochemistry and Molecular Biology, University of North Dakota, Grand Forks, ND 58202, USA

**Keywords:** PDGFR, c-MYC, JAG2, miR-1280, medulloblastoma

## Abstract

Over-expression of PDGF receptors (PDGFRs) has been previously implicated in high-risk medulloblastoma (MB) pathogenesis. However, the exact biological functions of PDGFRα and PDGFRβ signaling in MB biology remain poorly understood. Here, we report the subgroup specific expression of PDGFRα and PDGFRβ and their associated biological pathways in MB tumors. c-MYC, a downstream target of PDGFRβ but not PDGFRα, is involved in PDGFRβ signaling associated with cell proliferation, cell death, and invasion. Concurrent inhibition of PDGFRβ and c-MYC blocks MB cell proliferation and migration synergistically. Integrated analysis of miRNA and miRNA targets regulated by both PDGFRβ and c-MYC reveals that increased expression of JAG2, a target of miR-1280, is associated with high metastatic dissemination at diagnosis and a poor outcome in MB patients. Our study may resolve the controversy on the role of PDGFRs in MB and unveils JAG2 as a key downstream effector of a PDGFRβ-driven signaling cascade and a potential therapeutic target.

## INTRODUCTION

Medulloblastoma (MB) is the most frequently diagnosed malignant pediatric brain tumor. Approximately 30% of patients with MB are resistant to therapies and prone to develop metastasis [1–9]. Several earlier studies have shown that over-expression and/or over-activation of certain genes such as PDGFRs and c-MYC in the tumor tissues of MB patients are correlated with an aggressive tumor phenotype and poor prognosis [10–14].

The activation of PDGFR signaling initiates events that culminate in cell proliferation, survival, and migration [15–19]. A plethora of evidence shows that the MB cells with abnormal PDGFR signaling, attributable to the over-expression of PDGFRs or their ligands [11, 12, 20, 21], or mutation in PDGFRα, are linked to metastatic disease [19]. PDGFRα was initially found to be highly expressed in metastatic MBs, and it was further proposed to be a therapeutic target for metastatic MB based on the results that metastatic MB cells lost their metastatic phenotypes (reduced capabilities on adhesion and migration *in vitro*) upon blockade of PDGFRα signaling using a PDGFRα-neutralizing antibody and a MAP2K1/2 inhibitor [11, 20]. These results led to the proposal by MacDonald and coworkers that PDGFRα is a bona fide therapeutic target for metastatic MB [11, 20]. However, in subsequent studies, the PDGFRα probe-set used in the microarray analysis by MacDonald and his coworkers was shown to detect PDGFRβ [12]. The controversy regarding the roles of PDGFRα and PDGFRβ in MB has continued, i.e., PDGFRα was deregulated in both the primary and metastatic tumors in a sleeping beauty mouse model of MB [22]. Taking earlier and more recent results as a whole, it is evident that the controversy lingers and the dispute remains unresolved.

MB tumors are highly heterogeneous. Based on their molecular and clinical characteristics, at least four subgroups, WNT, SHH, Group 3, and Group 4 exist [14, 23–33]. Among these subgroups, the preferential survival rates of MB patients from good to poor are: WNT> SHH / Group 4>Group 3 [29, 34]; however, metastatic MBs are found in all subgroups. As yet, the role of PDGFR-mediated signaling has not been examined in the context of MB subgroups, and whether PDGFRα and PDGFRβ could initiate convergent or divergent events in MB remains to be determined.

c-MYC is a proto-oncogene encoding a transcription factor that controls multiple cellular events such as proliferation [35, 36], cell cycle [37–39], and apoptosis [40–43] by regulating the expression of its target genes. Over-expression of c-MYC promotes tumorigenesis while inhibition of c-MYC reduces tumor growth *in vitro* and *in vivo* [10, 13]. It has been shown that over-expression or oncogenic activation of c-MYC in MB may be also linked to an aggressive phenotype, and MB patients with elevated levels of c-MYC often have poor outcomes [10, 13, 14, 44, 45]. Inhibition of c-MYC using either siRNA or pharmacological intervention has been shown to limit tumor growth *in vitro* [43, 46–49]. These studies suggest that c-MYC plays a crucial role in MB biology.

Notch signaling, one of major determinants regulating cell differentiation [50], is a critical pathway regulating stem cell differentiation and tumor progression [51–54]. Abnormal activation of Notch pathway was demonstrated to induce tumor formation [50, 55]. A few studies indicate that Notch signaling may play a role in MB progression [53]; however, whether the regulation of Notch signaling by PDGFR in MB has not been reported.

In this study, we analyzed the expression levels of PDGFRα and PDGFRβ in primary MB for their associated gene signatures. We further used MB cells to elucidate their individual functions on cell proliferation, migration, and invasion. Moreover, by combining miRNA profiling with bioinformatics-aided target prediction complemented by experimental validation, we identified a potential novel therapeutic target, JAG2, which appears to act as a downstream target of the PDGFRβ-c-MYC signaling pathway. We further determined the expression levels of JAG2 in MB tissues for its prognostic value.

## RESULTS

### Expression of PDGFRα and PDGFRβ is associated with different prognosis in patients with MB

**Figure 1:**
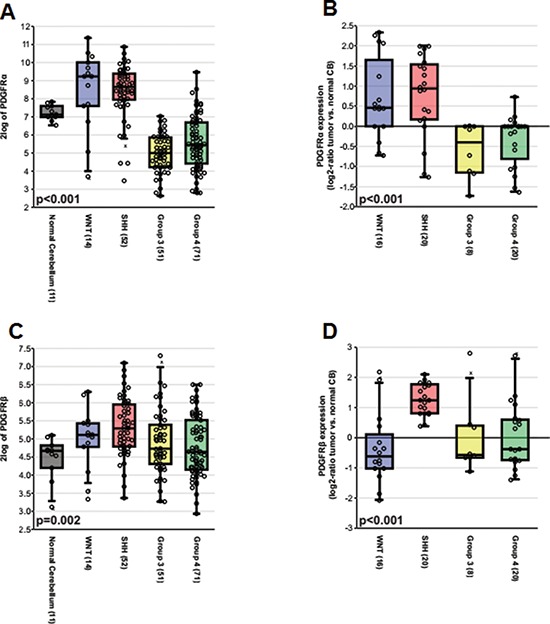
The subgroup specific expression of PDGFRα and PDGFRβ in primary MB **(A)** Boxplot showing PDGFRα expression in normal adult cerebellar samples and MB subgroups based on the Boston cohort (*n* = 199). **(B)** Relative expression of PDGFRα as a log2-ratio compared to a pool of normal cerebellar samples according to MB subgroups based on the Heidelberg cohort (*n* = 64). **(C)** Boxplot showing PDGFRβ expression in normal adult cerebellar samples and MB subgroups based on the Boston cohort. **(D)** Relative expression of PDGFRβ as a log2-ratio compared to a pool of normal cerebellar samples according to MB subgroups based on the Heidelberg cohort.

To define the biological roles of PDGFRs in MB, we analyzed the subgroup dependent mRNA levels of PDGFRα and PDGFRβ in two independent, non-overlapping gene expression profiling data sets [29, 56, 57]. As shown in Figure 1A, 1B, 1C, 1D and Table S1, the expression of PDGFRα was elevated in WNT and SHH subgroups (*p* < 0.001), while high levels of PDGFRβ were found in a subset of tumors from all subgroups, particularly high in SHH tumors (*p* < 0.001). We further analyzed the expression patterns in 3 sets of data and obtained similar results (Figure S1) [32, 58, 59]. Our previous studies revealed that patient with WNT MB has a better outcome than the one with SHH / Group 4 and Group 3 MBs [29, 34]. Our results suggest that expression of PDGFRα and PDGFRβ may be associated with the differences in prognosis.

We next searched for the molecular signatures of PDGFRα, PDGFRβ, and c-MYC in MBs using the R2 software (http://r2.amc.nl) by assessing the correlations of genes in major pathways with cellular functions in five cohorts of MBs previously determined by microarray from at least more than 45 samples containing all 4 subgroups of clinical MBs [29, 32, 33, 59, 60]. By analyzing the KEGG (Kyoto Encyclopedia of Genes and Genomes) pathway annotation in these data sets, we revealed that several pathways were significantly associated with PDGFRα and PDGFRβ expression, respectively, in the five separate tumor cohorts. As shown in Table 1, Supplemental Tables S2, S3, both the expression of PDGFRα and PDGFRβ in MB tumors was associated with signatures related to ‘ECM receptor interaction’, ‘Focal adhesion’, and ‘Pathways in cancer’. Notably, distinct signaling pathways for PDGFRα and PDGFRβ were also identified. For instance, ‘Wnt signaling pathway’, ‘Hedgehog signaling pathway’, and ‘Hippo signaling pathway’ were only associated with PDGFRα expression; while ‘Cell adhesion molecules_CAMs’, ‘Apoptosis’, ‘NFĸB signaling pathway’, and ‘Cytokine_cytokine receptor interaction’ were only associated with PDGFRβ expression. These data suggest that PDGFRs regulate distinct cellular functions in MB including cell proliferation, cell death, and cellular mobility.

**Table 1:** Pathway analysis of genes co-expressed with PDGFRα, PDGFRβ, and c-MYC in MB tumors

PDGFRα		PDGFRβ		c-MYC	
Antigen_processing_and_presentation	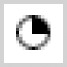	Adherens_junction	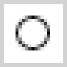	Ubiquitin_mediated_proteolysis	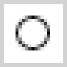
Arachidonic_acid_metabolism	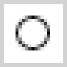	Amoebiasis*	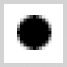	Spliceosome	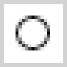
Basal_cell_carcinoma	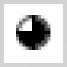	Antigen_processing_and_presentation	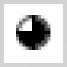	Ribosome_biogenesis_in_eukaryotes	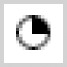
ECM_receptor_interaction	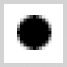	Apoptosis*	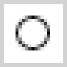	Ribosome	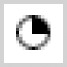
Focal_adhesion	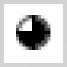	Cell_adhesion_molecules__CAMs*	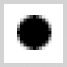	Protein_processing_in_endoplasmic_reticulum*	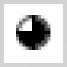
Hedgehog_signaling_pathway*	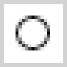	Complement_and_coagulation_cascades	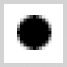	Protein_export*	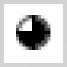
Hippo_signaling_pathway	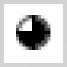	Cytokine_cytokine_receptor_interaction*	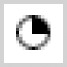	Olfactory_transduction*	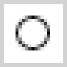
Insulin_secretion	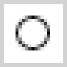	ECM_receptor_interaction	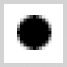	Neuroactive_ligand_receptor_interaction	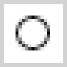
Leukocyte_transendothelial_migration	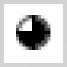	Focal_adhesion	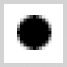	N_Glycan_biosynthesis*	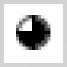
Lysosome	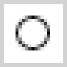	Hematopoietic_cell_lineage*	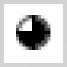	Measles*	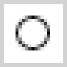
Neuroactive_ligand_receptor_interaction*	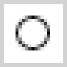	Inflammatory_bowel_disease__IBD_	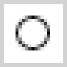	Jak_STAT_signaling_pathway*	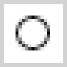
Pathways_in_cancer	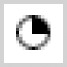	Insulin_secretion	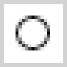	Glycolysis_Gluconeogenesis*	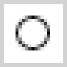
Phagosome	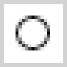	Leukocyte_transendothelial_migration	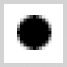	Cytokine_cytokine_receptor_interaction	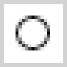
PI3K_Akt_signaling_pathway	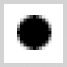	MicroRNAs_in_cancer*	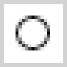		
Protein_digestion_and_absorption	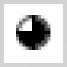	NF_kappa_B_signaling_pathway*	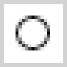		
Proteoglycans_in_cancer	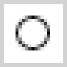	Pathways_in_cancer	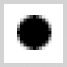		
Regulation_of_actin_cytoskeleton	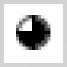	Phagosome	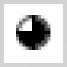		
Ribosome*	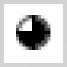	PI3K_Akt_signaling_pathway	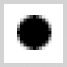		
Staphylococcus_aureus_infection	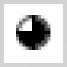	Protein_digestion_and_absorption	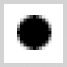		
Sulfur_metabolism*	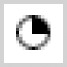	Proteoglycans_in_cancer	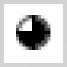		
Wnt_signaling_pathway*	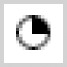	Regulation_of_actin_cytoskeleton	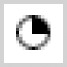		
		Small_cell_lung_cancer*	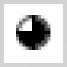		
		Staphylococcus_aureus_infection	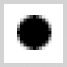		
		TNF_signaling_pathway*	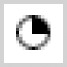		
		Viral_myocarditis	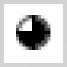		

The major pathways identified from human MBs based on the highly co-expressed genes.

* Genes in the pathways are specifically co-expressed with PDGFRα, or PDGFRβ, or c-MYC in MBs

The frequency of pathways found in 5 sets of data are marked as following: 2 out of 5: 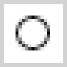 3 out of 5: 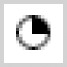 4 out of 5: 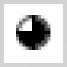 5 out of 5: 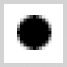

### PDGFRβ instead of PDGFRα promotes MB progression

**Figure 2:**
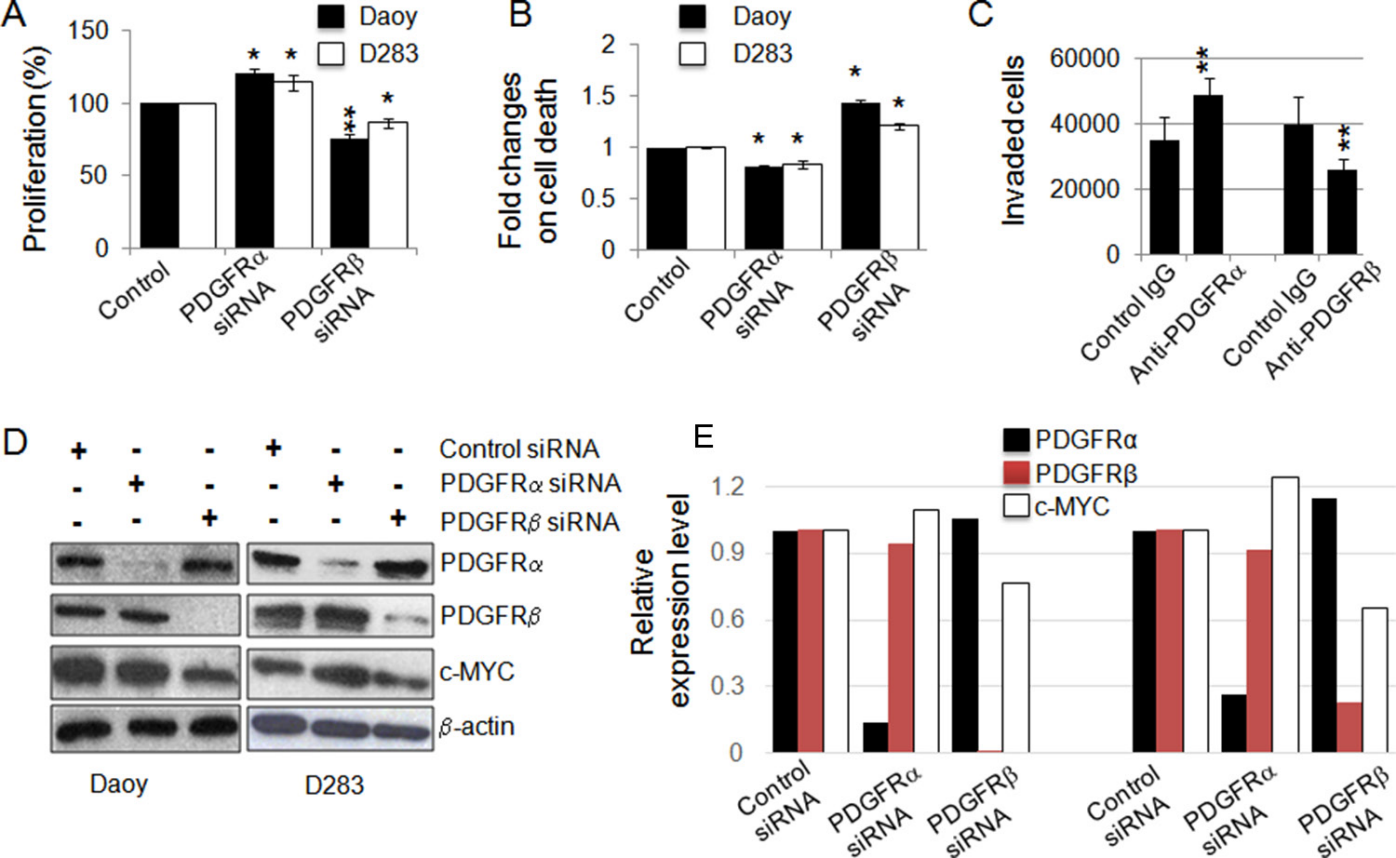
PDGFRs have distinct cellular functions and PDGFRβ regulates c-MYC **(A)** Effect of siRNA-mediated knockdown of PDGFRα and PDGFRβ in MB cells on cell proliferation. After 48 h of transfection, the rates of cell proliferation were determined using MTS assay; **(B)** siRNA-mediated knockdown of PDGFRα and PDGFRβ in MB cells on cell death. PDGFRα and PDGFRβ in MB cells were knocked down using siRNA and the rate of cell death was determined by LDH at 48 h; **(C)** Daoy cell migration/invasion was assessed in the presence of a control antibody, PDGFR neutralizing antibodies (mouse IgG as control for anti-PDGFRα, goat IgG as control for anti-PDGFRβ) as described in the Materials and Methods. *p < 0.05, **p < 0.01 (paired Student's *t*-test, sample vs. control); **(D)** PDGFRα and PDGFRβ signaling differentially regulates the expression of c-MYC in MB cells. PDGFRα and PDGFRβ in MB cells were knocked down using siRNAs. After 48 h of siRNA transfection, cells were harvested as protein lysates for Western blotting analysis; **(E)** The relative levels of PDGFRs and c-MYC in response to the siRNA treatments were calculated from the gel images of (D).

The distinct expression patterns of PDGFRα and PDGFRβ in MB subgroups and the association of distinct signaling pathways of PDGFRs in MBs led us to hypothesize that PDGFRα and PDGFRβ have distinct roles in MB progression. To functionally characterize the biological impact of these signaling events induced by the two PDGFRs, we assessed the effects on Daoy and D283 MB cells in cell proliferation and cell death in response to siRNA knockdown either PDGFRα or PDGFRβ. PDGFRβ knockdown resulted in decreased cell proliferation and increased cell death (*p* < 0.01; *p* < 0.01, respectively), while treatment with PDGFRα siRNA showed an increased cell proliferation and reduced cell death (*p* < 0.05; *p* < 0.05, respectively) (Figure 2A, 2B) in both Daoy and D283 cells. We also checked cell invasion under conditions of PDGFRα and PDGFRβ blockade using their respective neutralizing antibodies. We observed that interference with PDGFRα signaling promoted invasion, while disrupted PDGFRβ signaling inhibited invasion in Daoy cells (Figure 2C, S2). These results lead to the conclusion that PDGFRβ but not PDGFRα is a critical element to promote aggressive behavior of MBs. To understand the distinct cellular functions elicited by PDGFRα and PDGFRβ in MB cells, we further analyzed the expression of c-MYC, a previously defined PDGF response gene as a key determinant involved in cell proliferation [61–63]. In cells featuring siRNA-mediated knockdown of PDGFRα or PDGFRβ, we found that c-MYC expression was reduced in PDGFRβ siRNA-treated but not PDGFRα siRNA-treated cells, suggesting that c-MYC may be a downstream target of PDGFRβ partially contributing to the differential effects initiated by PDGFRα and PDGFRβ (Figure 2D, 2E).

### Co-targeting PDGFRβ and c-MYC expression reduces MB cell proliferation

**Figure 3:**
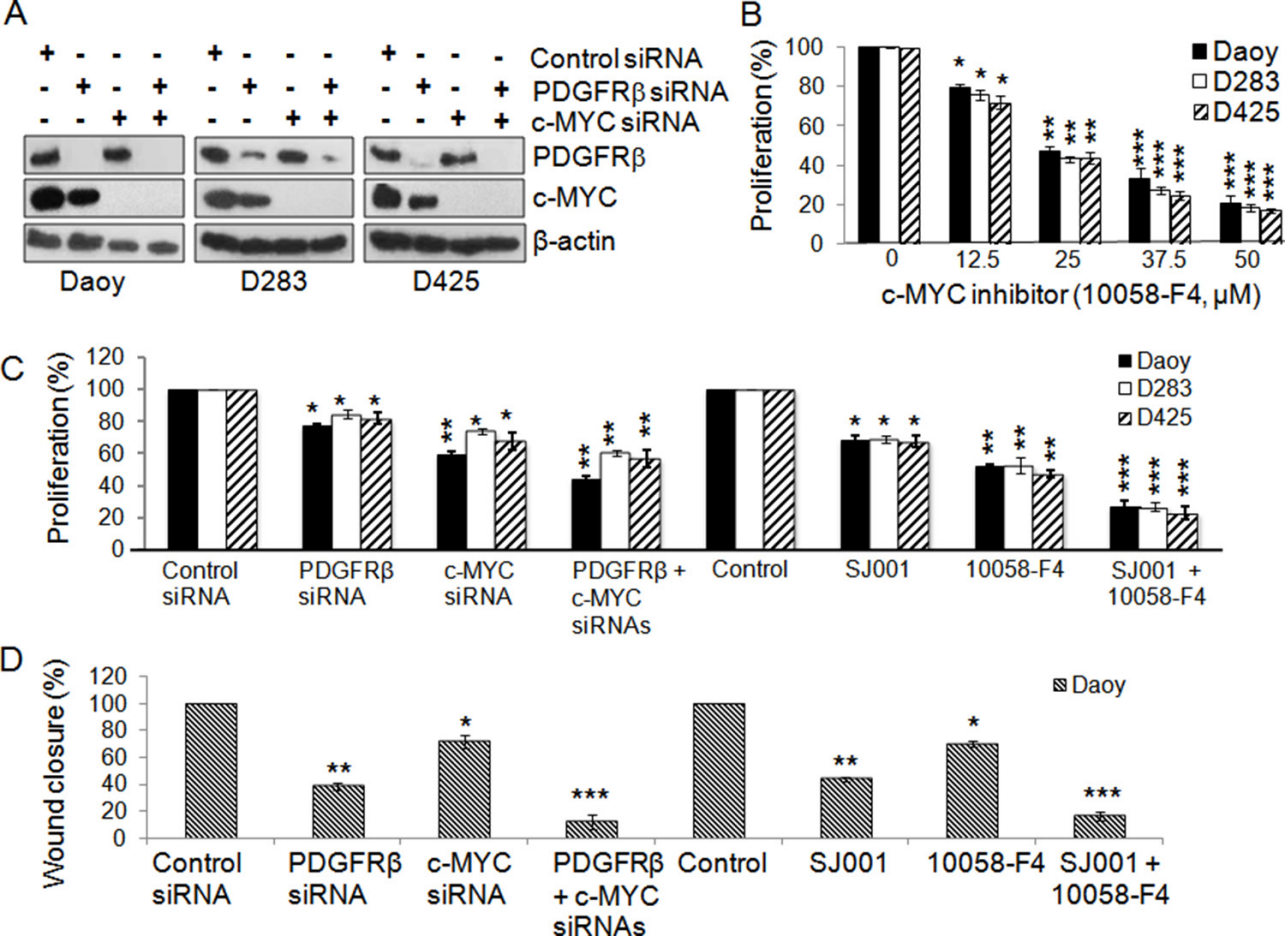
Co-inhibition of PDGFRβ and c-MYC suppresses MB cell proliferation and migration MB cells were transfected with gene-specific siRNAs for PDGFRβ and c-MYC and also with PDGFR and c-MYC specific inhibitors alone or in combination for 48 h. **(A)** Confirmation of specific gene knockdown by Western blotting analysis. β-actin was used as the loading control. **(B)** The effects of c-MYC inhibitor 10058-F4 on MB cell proliferation. **(C)** The effects of siRNAs and inhibitors on MB cell proliferation were determined using MTS. *p < 0.05, **p < 0.01 (paired *t*-test, sample vs. control). **(D)** The effects of co-inhibiting PDGFRβ and c-MYC on MB cell migration. Daoy cells were transfected with gene specific siRNAs for PDGFRβ and c-MYC and also with PDGFR and c-MYC specific inhibitors alone or in combination for 36 h. Treated cells were then detached and re-distributed in equal amounts in a 48-well plate before a linear wound was made. The image was captured immediately after that an artificial wound was made at 0^th^ h and also at 24^th^ h (Figures S3a, S3b). Quantified results were calculated from the images. Percentage wound closure shows the migration rate in PDGFRβ^KD^, c-MYC^KD^ or PDGFRβ^KD^c-MYC^KD^ cells when compared to control sample, *p < 0.05, **p < 0.01, ***p < 0.001 (paired *t*-test, sample vs. control).

Analysis of MBs using high-resolution DNA copy number profiling showed that c-MYC amplification was mainly found in Group 3 MBs (25% of MBs) [60]; while Grotzer et al. demonstrated by RT-PCR using a cohort of 26 MBs that all MBs express c-MYC [44]. We thus also analyzed the genes in the pathways co-expressed with c-MYC in the 5 data sets [29, 32, 33, 59, 60] and our data suggest that c-MYC and PDGFRβ activate distinct signaling pathways in MBs (Table 1, Supplemental Tables S3, S4). Based on previous reports that c-MYC over-expression is critical for MB progression [10, 13], we reasoned that co-targeting both PDGFRβ and c-MYC could maximize the suppression of MB progression, especially in SHH and Group 3 MBs. On the basis of phenotypes and molecular features, Daoy may be regarded as a SHH tumor, while D283 and D425 are considered as Group 4 and Group 3 MB tumors, respectively [14, 23–33, 64]. All 3 cell lines with detectable PDGFRs and c-MYC (Figure 3A) were used to target both PDGFRβ and c-MYC simultaneously using siRNAs against PDGFRβ and c-MYC, and also pharmacological inhibitors, SJ001 (a novel PDGFR inhibitor, also called cambogin) [65] and 10058-F4 (it inhibits the c-MYC-Max interaction) [46–48]. The specificity and efficacy of siRNA knockdown of PDGFRβ or c-MYC or both were confirmed by Western blotting analysis (Figure 3A). The results show that blockade of PDGFRβ and c-MYC signaling using either RNA interference or pharmacological intervention inhibited both MB cell proliferation and migration (Figure 3B, 3D). Although in Figure 2D, we show that c-MYC is partially regulated by PDGFRβ signaling, the observation of that PDGFRβ signaling affected cell migration more significantly than proliferation, whereas c-MYC signaling mainly contributed to suppression of cell proliferation, indicating that PDGFRβ and c-MYC act on different pathways. Notably, SJ001 is able to inhibit both PDGFRα and PDGFRβ [65], indicating that PDGFRβ may have a decisive role on cell proliferation and migration in MB when both PDGFRs are repressed simultaneously. Co-targeting of PDGFRβ and c-MYC using either gene specific siRNAs or pharmacological inhibitors potentiated the effects on cell proliferation and migration, as shown by synergism in suppression of cell proliferation and migration compared to blockade of either c-MYC or PDGFRβ alone (Figure 3C, 3D).

### miR-1280 expression is regulated by PDGFRβ and c-MYC, and functionally important for MB cells

**Figure 4:**
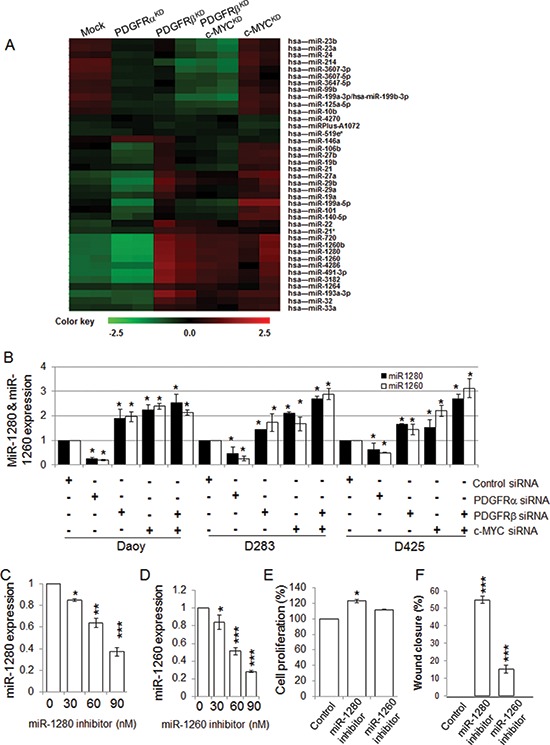
PDGFRβ and c-MYC differentially regulate miRNA expression in MB cells **(A)** Heat map represents the differentially regulated miRNAs by Control (Mock), PDGFRα^KD^, PDGFRβ^KD^, c-MYC^KD^ or PDGFRβ^KD^c-MYC^KD^ cells. The normalized log ratio values were used for the analysis. The clustering was performed on all samples, and the highly differentially regulated 39 miRNAs were selected. Each row represents a miRNA and each column represents a sample. The color scale illustrates the relative expression levels of miRNAs. Green color represents an expression level below the reference channel, and red color represents the expression higher than the reference. **(B)** The expression levels of miRNAs regulated by PDGFRα, PDGFRβ, and c-MYC in MB cell lines. MB cells were transfected with gene specific siRNAs for PDGFRα, PDGFRβ, and c-MYC alone or in combination of PDGFRβ and c-MYC for 24 h. *p* < 0.05 was considered to be statistically significant. **(C)** miR-1280 inhibitor suppresses miR-1280 expression in Daoy cells. PDGFRβ^KD^ Daoy cells were transfected with control or increasing concentrations of miR-1280 specific inhibitor using Lipofectamineltx in opti-MEM reduced serum medium following the manufacturer's instruction for 4 h. The cells were then fed with equal volume of MEM medium with 10% FBS. After 24 h, total RNA isolated was subjected to TaqMan microRNA assay to verify for specific inhibition of miRNAs. Data are presented as mean values (*n* = 3) ± standard deviation. Differences between 2 groups were analyzed using Student's *t*-test. *p* < 0.05 was considered to be statistically significant. **(D)** miR-1260 inhibitor suppresses miR-1260 expression in Daoy cells. **(E)** The effects of miR-1280 and miR-1260 on MB cell proliferation. PDGFRβ^KD^ Daoy cells were treated with control or miR-1280 inhibitor or miR-1260 inhibitor. The effects on cell proliferation were determined by MTS assay. **(F)** The effects of miR-1280 and miR-1260 on PDGFRβ^KD^ Daoy cell migration. PDGFRβ^KD^ Daoy cells were treated with control or miR-1280 inhibitor or miR-1260 inhibitor. The treated cells were then detached and re-distributed in equal amounts in a 48-well plate before a linear wound was made. The image was captured immediately after an artificial wound was made at 0^th^ h and also at 24^th^ h (Figure S4a). Quantified results were calculated from the images. The treated samples were compared to control sample, *p < 0.05, **p < 0.01, ***p < 0.001 (paired *t*-test, sample vs. control).

To find the common targets which are optimally responsible for both migration/invasion and proliferation, we explored the involvement of miRNAs in PDGFRα, PDGFRβ, and c-MYC signaling in MB cells by determining the miRNA profiles of Daoy cells without (Mock) and with knockdown of PDGFRα (PDGFRα^KD^), PDGFRβ (PDGFRβ^KD^), c-MYC (c-MYC^KD^), and both PDGFRβ and c-MYC (PDGFRβ^KD^c-MYC^KD^) using the miRCURY LNA^TM^ microRNA Array (6th Gen) platform. We observed that knockdown of PDGFRα had little effect on miRNA expression, while knockdown of either PDGFRβ or c-MYC markedly changed the miRNA profiles (Figure 4A). Among 1497 tested miRNAs, 159 miRNAs were modulated by PDGFRβ (57 up-regulated and 102 down-regulated); 125 miRNAs were altered by c-MYC (76 up-regulated and 49 down-regulated), and 22 miRNAs responded to both PDGFRβ and c-MYC. Comparative analysis of the respective miRNA profiles revealed that among 39 highly regulated miRNAs, a subset of miRNAs including miR-1280 and miR-1260 was concordantly regulated by PDGFRβ and c-MYC (Figure 4A). Next we analyzed the expression levels of miR-1280 and miR-1260 (two highly regulated miRNAs by both PDGFR and c-MYC) by real-time RT-PCR upon knockdown of PDGFRα and PDGFRβ, each separately or both combined, in three MB cell lines (Daoy, D283, and D425). The results agreed with the data generated by miRNA profiling analysis (Figure 4B). To determine the biological functions of miR-1280 and miR-1260 in MB cells, PDGFRβ^KD^ Daoy cells were treated with either a miR-1280 inhibitor or a miR-1260 inhibitor, and cells were then analyzed for miR-1280 or miR-1260 expression, cell proliferation, and migration. We found that both miR-1280 and miR-1260 inhibitors down-regulated the expression of miR-1280 and miR-1260 (Figure 4C, 4D), respectively, in PDGFRβ^KD^ Daoy cells. Notably, treatment with the miR-1280 inhibitor restored the cellular function of PDGFRβ^KD^ cells; while the miR-1260 inhibitor had less pronounced effects on both cell proliferation and migration. Thus, we selected miR-1280 for the further investigation. In response to miR-1280 inhibition, PDGFRβ^KD^ cells showed 15% and 55% increases in cell proliferation and in cell migration (Figure 4E, 4F), respectively. Taken together, these results provide a strong support that PDGFRα and PDGFRβ regulate different molecular downstream targets, resulting in distinct functional roles for MB progression.

### Identification of JAG2 as a potential new MB therapeutic target regulated by PDGFRβ and c-MYC

**Figure 5:**
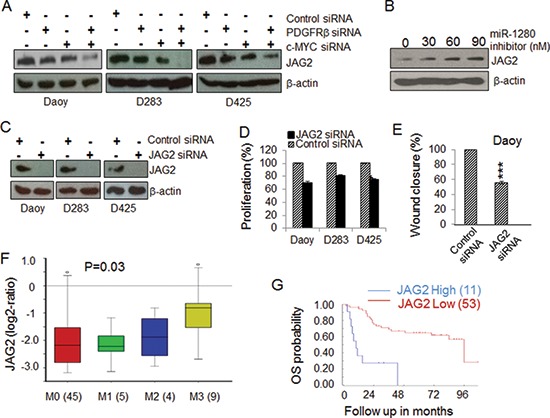
Identification of JAG2 as a potential target of MB **(A)** The expression of JAG2 in MB cells is regulated by PDGFRβ and c-MYC. MB cells were transfected with gene specific siRNAs against PDGFRβ and/or c-MYC for 48 h. Protein lysates extracted from treated samples were used for the expression levels of JAG2 by Western blotting. β-actin was used as a loading control. **(B)** The expression levels of JAG2 are regulated by a miR-1280 inhibitor. PDGFRβ^KD^ Daoy cells were transfected with increasing concentrations of a miR-1280 specific inhibitor. After 48 h, protein lysates extracted were subjected to Western blotting analysis to check for change in expression of JAG2 protein. β-actin was used as a loading control. **(C)** Confirmation of specific knockdown JAG2 by Western blotting analysis. β-actin was used as the loading control. **(D)** The effects of JAG2 siRNA on MB cell proliferation. MB cells were transfected with control or JAG2 specific siRNAs for 48 h. Cell proliferation was determined using MTS. **(E)** The effects of JAG2 on MB cell migration. PDGFR^KD^ Daoy cells were treated with control or JAG2 siRNAs for 36 h and then detached and re-distributed in equal amounts in a 48-well plate before a linear wound was made. The image was captured immediately after an artificial wound was made at 0^th^ h and also at 24^th^ h (Supplemental Figure S4B). Quantified results were calculated from the images. The significance of JAG2 siRNAs on MB cell migration were analyzed using paired *t*-test, sample vs. control (*p < 0.05, **p < 0.01). **(F)** The expression of JAG2 in MB tissues of different stages of metastasis (45 patients at M0 stage, 5 patients at M1 stage, 4 patients at M2 stage, and 9 patients at M3 stage); **(G)** Kaplan-Meier plots of overall survival (OS) time according to JAG2 levels in MB patients.

To further understand the miRNA network regulated by PDGFRβ and c-MYC, we used a miRNA target prediction strategy as previously outlined [66, 67] to analyze the potential targets of the highly regulated miRNAs. Based on the number of potential interaction sites between miRNA and its potential targets, the score of sequence alignment at the 3′ UTR of the potential target gene and available literature regarding the function of the potential targets, we identified that JAG2 is a potential target regulated by miRNAs under the control of both PDGFRβ and c-MYC. These results suggest that PDGFRβ and c-MYC likely modulate genes related to cell proliferation and survival via certain miRNAs and their targets, including JAG2 as a target of miR-1280. To further test and validate the results obtained from bioinformatics aided identification of miRNA target, we treated MB cells with PDGFRβ and c-MYC siRNAs alone or in combination and then analyzed the expression levels of JAG2 protein by Western blotting analysis. A decrease in JAG2 expression occurred in MB cells lacking either PDGFRβ or c-MYC. An even more significant reduction of JAG2 protein levels was observed in MB cells lacking both PDGFRβ and c-MYC compared to either control or knockdown of PDGFRβ and c-MYC alone (Figure 5A). These results indicate that both PDGFRβ and c-MYC regulate the expression of JAG2 via miR-1280 in MB cells. This conclusion is further supported by the fact that the level of JAG2 increased markedly in the cells treated with a miR-1280 inhibitor compared to the control (Figure 5B). To further elucidate the role of JAG2 in MB biology, JAG2 was specifically knocked down using siRNAs in all three MB cell lines (Figure 5C), and its effects on cell proliferation and migration were analyzed using MTS and wound healing assay, respectively. The results showed that JAG2 knockdown in MB cells reduced cell proliferation (*p* < 0.01) and migration (*p* < 0.001) (Figure 5D, 5E). To determine the clinical significance of JAG2 in MB, we analyzed the prognostic values of JAG2 expression levels in 64 MB samples. We observed increased expression levels of JAG2 in metastatic MB tumors (Figure 5F, *p* < 0.05) and the expression levels of JAG2 correlated with poor prognosis outcomes (Figure 5G, *p* < 0.001).

## DISCUSSION

Over-expression of PDGFRs has been considered as a hallmark feature of MB metastasis and thus as therapeutic targets in high-risk MBs [10–14]. In this study, we found that the expression of PDGFRα and PDGFRβ in MBs is subgroup specific and associated with distinct molecular signatures; and only elevated level of PDGFRβ is linked with an aggressive phenotype of MB *in vitro* experiments. We present data showing that PDGFRα and PDGFRβ differentially regulate MB cellular functions - with PDGFRα limiting and PDGFRβ promoting cell proliferation, survival, and migration/invasion. Through targeting PDGFRβ and c-MYC, we revealed that the biological effects of PDGFRβ appear to be orchestrated by novel mediators including the miR-1280-JAG2 axis, which enhanced MB proliferation and migration *in vitro*. We further showed that the expression of JAG2 is correlated with the stages of MB metastasis and associated with a poor outcome in MB patients, indicating that JAG2 is a novel potential target. Thus our mechanistic studies on PDGFRβ promoting MB growth and migration via miRNAs and miRNA targets provide new insight that may help resolve the long-standing controversy on the role of PDGFRs in MB.

The notion that PDGFRα and PDGFRβ contain well-conserved structure and display largely redundant functions has been documented and generally accepted. However, in this study, we present data showing that PDGFRα and PDGFRβ play distinct roles in MB cells by differentially regulating MB cellular functions. Notably, structure-function analysis of the two PDGFRs shows that although they share 70% homologues in the N-termini and 80% in the C-termini of the kinase domain [68], significant differences exist in their ligand binding domain (31% identical) and a sub-domain located at the C-terminal region (27–28% homologues). These dissimilar structural features presumably can account for, at least in part, how the two receptors show differential ligand binding specificity and affinity and additionally, interaction with unique target protein sets to mediate starkly distinct functions *in vitro* [69] and *in vivo* [68, 70, 71].

Oncogenic activation of the c-MYC gene is commonly observed in MBs [13, 14, 72, 73] and over-expression of c-MYC is one of the critical features of Group 3 MBs [25, 60]. The subset of MB patients with 17p loss and higher levels of c-MYC is characterized by shorter survival [14]. Notably, majority of the MB cell lines established from pediatric MB patients express high levels of c-MYC [13, 72]. Based on our current study, the over-expression of c-MYC in MB could be partially due to abnormal PDGFR signaling because c-MYC is a PDGFRβ downstream target (Figure 2D). Although c-MYC expression is partially regulated by PDGFRβ, c-MYC and PDGFRβ regulate cellular functions differently, since PDGFRβ siRNA or inhibitor had a greater inhibitory effect on migration, while c-MYC siRNA or inhibitor primarily blocked MB cell proliferation. Importantly, only blockade of PDGFRβ showed significant effects on limiting cell invasion (Figure 2C), suggesting that PDGFRβ may play a more critical role in MB invasion. Although our current data from patients with MB that high levels of PDGFRβ are mainly observed in SHH tumors, high levels of PDGFRβ also exist in other subgroups and all cell lines tested in this study (Figure 1D, 3A). Therefore, targeting both PDGFR and c-MYC might provide a novel therapeutic strategy for treating MB. Indeed, we have demonstrated that the combined inhibition of both PDGFRβ and c-MYC using either gene-specific siRNAs or pharmacological inhibitors showed additive inhibitory effects on both MB cell proliferation and migration compared to single knockdown of either PDGFRβ or c-MYC (Figure 3C, 3D).

**Figure 6:**
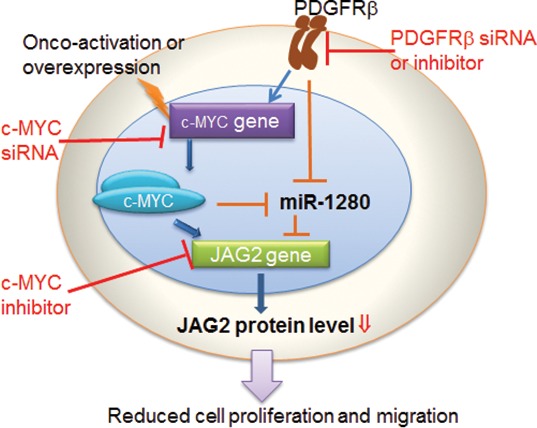
Schematic representation of pathways promoting MB progression by PDGFRβ and c-MYC, and the axis connecting PDGFRβ to JAG2, through c-MYC and miR-1280

MicroRNAs provide an important mechanism for modulating signaling pathways [74–78]. While analysis the PDGFR specific signatures in MB tumors, we noticed the results from 2 out of 5 data sets showing that PDGFRβ, but not PDGFRα, was associated with miRNA_in_cancer (Tables S2, S3). Our array knockdown analysis of individual PDGFRs also show that PDGFRα signaling has little effects on miRNA regulation in the tested cells (Figure 4A). Through miRNA profiling, target prediction and validation, we revealed that PDGFRβ and c-MYC may modulate MB biology via a set of highly regulated miRNAs (Figure 4A) and miRNA targets. Notably, Schopman et al. showed that miR-1280 might be a fragment of a tRNA based on their sequence similarity and annotation [79]. We first confirmed the expression of miR-1280 in different MB cell lines that responded to knockdown of PDGFRα, PDGFRβ or c-MYC. We further demonstrated that the miR-1280 inhibitor suppressed the expression of miR-1280 in MB cells (Figure 4C), and that this suppression had a more pronounced effect on cellular motility than proliferation (Figure 4E, 4F). Knockdown of either PDGFRβ, c-MYC alone or in combination provided evidence of an inverse correlation between the expression levels of miR-1280 and its expected target, JAG2 (Figure 4B, 5A). The expression of JAG2 in MB cells regulated by miR-1280 was further confirmed in studies using a miR-1280 inhibitor (Figure 5B). These results support the notion that JAG2 is a bona fide target of miR-1280. Furthermore, the effects of JAG2 siRNA phenocopied the functional effects of the miR-1280 inhibitor on Daoy cell proliferation and migration in the current study. Now, we have direct evidence that JAG2 is involved in PDGFRβ and c-MYC signaling, supporting the novel PDGFRβ-c-MYC-JAG2 regulatory axis in MB growth and migration (Figure 6). Our findings are in line with a recent paper indicating that Notch signaling and c-MYC signaling transduction are linked in MBs [80]. However, it further extends this report by suggesting an upstream regulatory role PDGFRβ in this signaling network and delineating that miR-1280 has an important role in the transcriptional regulation of JAG2. Further, here we demonstrate for the first time that JAG2 is regulated by PDGFRB and c-MYC and there is an immediate functional impact of JAG2 abrogation in MBs. Given that c-MYC is a well-known driver of oncogenesis in many models and cancer types, and that JAG2 is a c-MYC-regulated gene [81], the PDGFRβ-c-MYC-JAG2 pathways would be a logical and expected mechanism of MB carcinogenesis.

JAG2 is a transmembrane glycoprotein that binds to notch receptors regulating cell proliferation and differentiation in both normal and pathological conditions [50, 82, 83]. The significance of Notch signaling in MB has been demonstrated by employing inhibitors designed to suppress various key regulators playing important roles in the Notch pathway, wherein accompanying changes manifested as a reduction in cell proliferation and increase in apoptosis were observed [51–53], thus implicating Notch signaling as a target that may constitute an additional promising treatment strategy for MB patients. By inference, therefore, it may be proposed that co-targeting of Notch and PDGFR signaling should constitute a more promising effective therapeutic modality for MB, particularly since previous data show that Notch1 inhibition may not be sufficiently robust to achieve tumor control in a small cohort of pre-treated, relapsed MBs [84].

In conclusion, our results demonstrate that PDGFRβ, not PDGFRα, plays an essential role in MB biology. The promoting of MB progression by PDGFRβ and c-MYC could through their several downstream effectors, among which a set of miRNAs, e.g., miR-1280, and concomitantly up-regulates the expression of tumorigenic factors, e.g., JAG2 are important contributors. As the existence of PDGFRβ and c-MYC expression MBs, simultaneous inhibition of PDGFRβ and c-MYC signaling in MB cells synergistically suppresses both cell migration and cell proliferation. We further revealed that the expression of JAG2 is linked with MB metastasis and patients with a poor outcome. Finally, our results proffer the therapeutic tenet that co-targeting of PDGFRβ and c-MYC, or PDGFRβ and Notch signaling may represent novel therapeutic strategies for the treatment of MB.

## MATERIALS AND METHODS

### Analysis of PDGFRs, c-MYC, and JAG2 expression in primary MB

Using the R2 software (http://r2.amc.nl), we analyzed candidate gene expression levels in primary MBs and normal cerebellar samples, and correlated gene expression patterns with subgroup annotation to a recently published gene expression profiling study [29, 32, 57–59]. The specific pathways associated with the expression of PDGFRα, PDGFRβ, and c-MYC in MBs were analyzed in 5 available complete data sets in the R2 database using the KEGG pathway finder option. The pathways that show significant (*p* ≤ 0.01, chi-squared test) enrichment based on the genes co-expressed with PDGFRs were identified and specific pathways found in at least 2 of 5 data sets were ordered [29, 32, 33, 59, 60]. In addition to standard descriptive and graphical analyses, qualitative and quantitative association of variables was also evaluated by one-way ANOVA or the Mann-Whitney test, respectively. Survival was measured from the time of initial diagnosis to the date of death or the date of last follow up. Survival distribution was estimated according to the Kaplan-Meier method using optimal cut-off selection and log-rank statistics with Bonferroni based multiple testing corrections. P-values < 0.05 were considered to be statistically significant.

### Cell culture

Human MB cell lines, Daoy and D283 were purchased from American Type Culture Collection and D425 cells were a gift from Dr. Darell D. Bigner [85]. Daoy and D283 cells were maintained in modified Eagle's medium (MEM) containing 10% fetal bovine serum (FBS), 2 mM non-essential amino acids and 5 mM sodium pyruvate. D425 cells were maintained in Zinc-rich MEM medium containing 10% FBS and cultured in a 5% CO_2_ incubator at 37°C.

### RNA interferences

PDGFRα siRNA duplex (5′-GGAGGAUG AUGAUUCUGCCAUUAUA-3′ ), PDGFRβ siRNA duplex (5′-UCACGGAAAUAACUGAGAUCACCAU-3′ ), and control (mock) siRNA duplex (5′-ACAUCACGUACGCG GAAUACUUCGA-3′) were obtained from Invitrogen. JAG2 siRNA, a pool of 4 siRNA duplexes (5′-GCAAGGAAGCUGUGUGUAA-3′, 5′-GCGUGUGC CUUAAGGAGUA-3′, 5′-GAACGGCGCUCGCUGC UAU-3′, 5′-GGUCGUACUUGCACUCACA-3′ ) were purchased from Dharmacon. c-MYC siRNA is a pool of 3 different siRNA duplexes (5′-CCCAAGGUAGUUAUCCUUAtt-3′, 5′-GGAAACGAC GAGAACAGUUtt-3′, and 5′-CCUGAGCAAUCA CCUAUGAtt-3′ ) were purchased from Santa Cruz. Transfection of siRNA was performed using Lipofectamine™ (Invitrogen) according to the company's instructions.

### Cell proliferation

Cells (2 × 10^4^/well) were incubated in 70% MEM with 10% FBS, 30% Opti-MEM with 15 pM siRNA, or miRNA inhibitor (Applied Biosystems) and 0.25 μl/well of Lipofectamine (Invitrogen) in 96-well plates. For the experiments using inhibitors, cells were treated with either SJ001 (cambogin, 5 μM) [65] or 10058-F4 (an inhibitor that disrupts the c-MYC-Max interaction) [45–47] (Calbiochem) at various concentration as indicated in the figures. At 48 h post-treatment, cell proliferation rates were determined using a MTS assay (Promega).

### Lactate dehydrogenase (LDH) based cell death index

Cells (2 × 10^4^/well) were placed in MEM without penicillin and streptomycin a day before transfection in 24-well plates. After 48 h of siRNA transfection, culture media were harvested for assessment of released LDH using an LDH based toxicology assay kit (Sigma).

### RT-PCR and western blotting

Real-time RT-PCR and Western blotting were performed as described [69]. The antibodies were purchased from various manufacturers: anti-human PDGFRα rabbit antibody (Santa Cruz), anti-human PDGFRβ rabbit antibody (Epitomics), anti-human PDGFRα mouse neutralizing antibody (R&D), anti-human PDGFRβ goat neutralizing antibody (R&D), c-MYC rabbit antibody (Sigma), JAG2 rabbit antibody (Cell Signaling), β-actin mouse antibody (Sigma), secondary rabbit HRP-conjugated antibody (Bio-Rad), secondary mouse HRP-conjugate antibody (Sigma).

### Invasion and wound healing assay

Boyden chamber 24-well invasion assay kit was purchased from Calbiochem. Invasion assays were performed as previously described [69]. Wound healing assays were used to measure the rate of cell migration [86]. Daoy cells were treated with siRNA, or inhibitor as indicated in the Figures. Equal amount of scrambled siRNA or solvent served as a control. At 36 h, cells were detached, and equal number of cells was re-distributed in a 48-well plate. After 48 h incubation, an artificial wound was made using a 100 μl pipette tip by scraping across the bottom of the well. The medium was changed to remove all detached cells. Movement of cells into the wound area was captured by taking images at 0 and 24 h using a phase-contrast microscope (Olympus). Migration rates in percentage were calculated by comparing the width of the wound at 0 and 24 h in each sample against control cells. Wound healing assay was not performed on D283 and D425 cells as they are half adherent/half suspension cells. Experiments were performed in triplicate. The results are presented as percentage for wound healing.

### MicroRNA (miRNA) profiling

Control shRNA, PDGFRα-shRNA and PDGFRβ-shRNA plasmids were prepared using a vector (pRNAT-CMV3.2/Neo) from GenScript. The plasmids were introduced into Daoy cells followed by G418 selection. The control cells (harboring a mask control shRNA vector) and PDGFRβ^KD^ cells were used to prepare c-MYC^KD^ and PDGFRβ^KD^c-MYC^KD^ cells, respectively, using c-MYC specific siRNA. Total RNAs were isolated from control, PDGFRα, PDGFRβ^KD^, c-MYC^KD^, and PDGFRβ^KD^ c-MYC^KD^ cells using the miRCURY^TM^ RNA isolation kit (Exiqon) following the manufacturer's protocol. NanoDrop 1000 spectrophotometer (Thermo Scientific) and agarose gel electrophoresis were used to assess the quality of the RNA isolated. The samples were labeled using the miRCURY LNA^TM^ microRNA Hi-Power Labeling kit Hy3^TM^/Hy5^TM^ (Exiqon) and hybridized on the miRCURY LNA^TM^ microRNA Array (6^th^ Gen, Exiqon). Duplicate samples for each cell type were applied to the array analysis. The quantified signals were normalized (background corrected) using the global Lowess regression algorithm and the highly differentially regulated miRNAs were selected and presented in the heat map.

### Determination of miRNAs by Taqman PCR

Total RNA was isolated using TRI reagent (Sigma). One μg of total RNA was used to prepare miRNA specific cDNA using TaqMan^®^ microRNA reverse transcription kit (Applied Biosystems). One μl of this cDNA was used to perform qRT-PCR using 20x TaqMan^®^ MicroRNA assay along with TaqMan Universal PCR master mix (Applied Biosystems) to validate the regulated miRNAs in MB cells. RNU6B was chosen as an endogenous control. Experiments were performed in duplicate. PCR was performed using the following program: initial enzyme activation at 95°C for 10 min, denaturation at 95°C for 15s followed by annealing/extension at 60°C for 1 min for 40 cycles. Fold change obtained from Ct values using 2^−ΔΔ Ct^ methodology [87] was converted into logarithmic base 2 for statistical analysis. *p* < 0.05 was considered to be statistically significant.

## SUPPLEMENTARY FIGURES AND TABLES








