# Supplementary material for: A microRNA-1280/JAG2 network comprises a novel biological target in high-risk medulloblastoma
Source: Oncotarget. 2014 Dec 17;6(5):2709–24. doi: 10.18632/oncotarget.2779 (PMC4413612; doi:10.18632/oncotarget.2779)
Supplement: Supplementary file 1 [file oncotarget-06-2709-s001.pdf]

SUPPLEMENTARY FIGURES AND TABLES

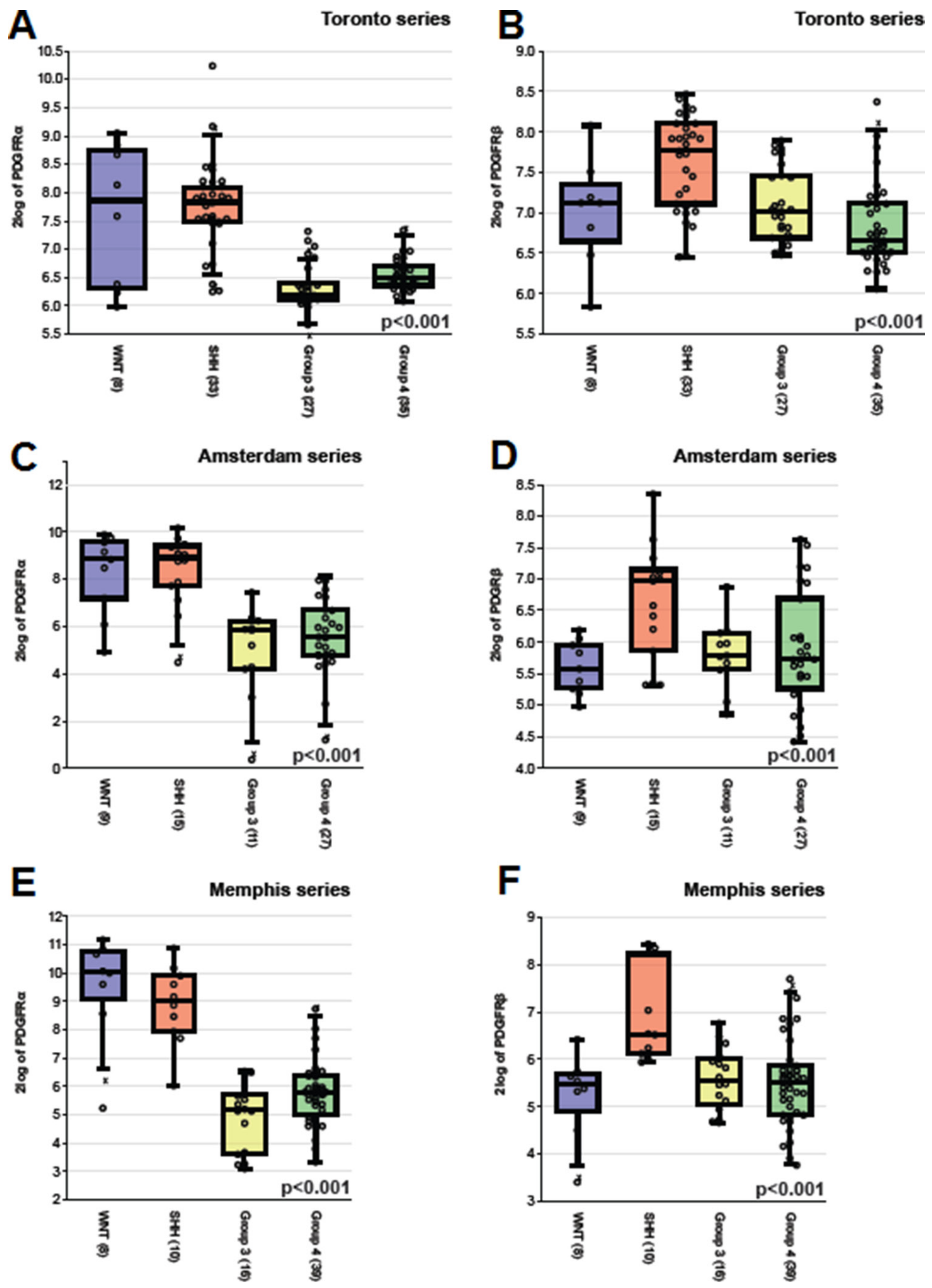

Supplementary Figure S1: The subgroup specific expression of PDGFRα and PDGFRβ in primary MB. Boxplot showing PDGFRα and PDGFRβ expression in three independent, non-overlapping gene expression profiling studies derived from Toronto (a/b), Amsterdam (c/d), and Memphis (e/f).

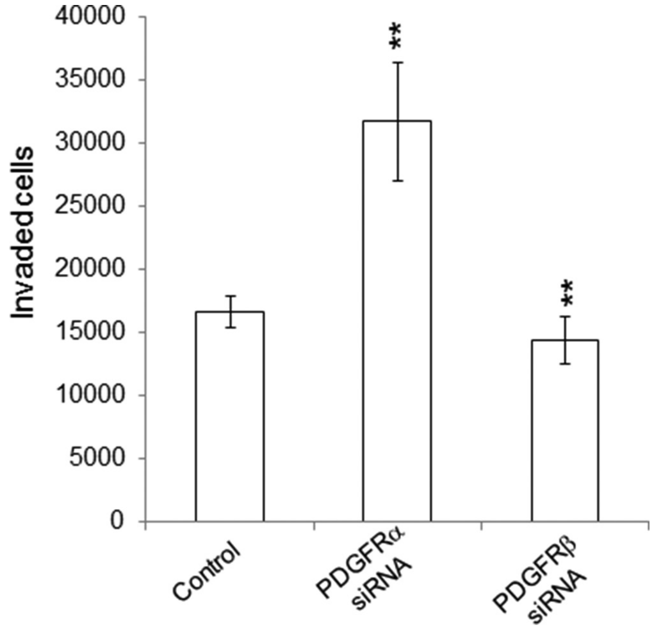

Supplementary Figure S2: The effect of PDGFR siRNA on Daoy cell invasion. A multi-well chamber-based assay was used to access the ability of cells to migrate through a membrane coated with matrigel. In the presence of control or PDGFR siRNAs, 2.5×10^4 Daoy cells were seeded in the upper chamber. 24 h later, cells that invaded or migrated to the lower chamber were labeled with Calcein AM. The labeled cells were measured for fluorescence with a fluorescence plate reader and set at an excitation wavelength of 485 nm and an emission wavelength of 520 nm. $**p < 0.05$ (paired *T* test, sample vs. control)

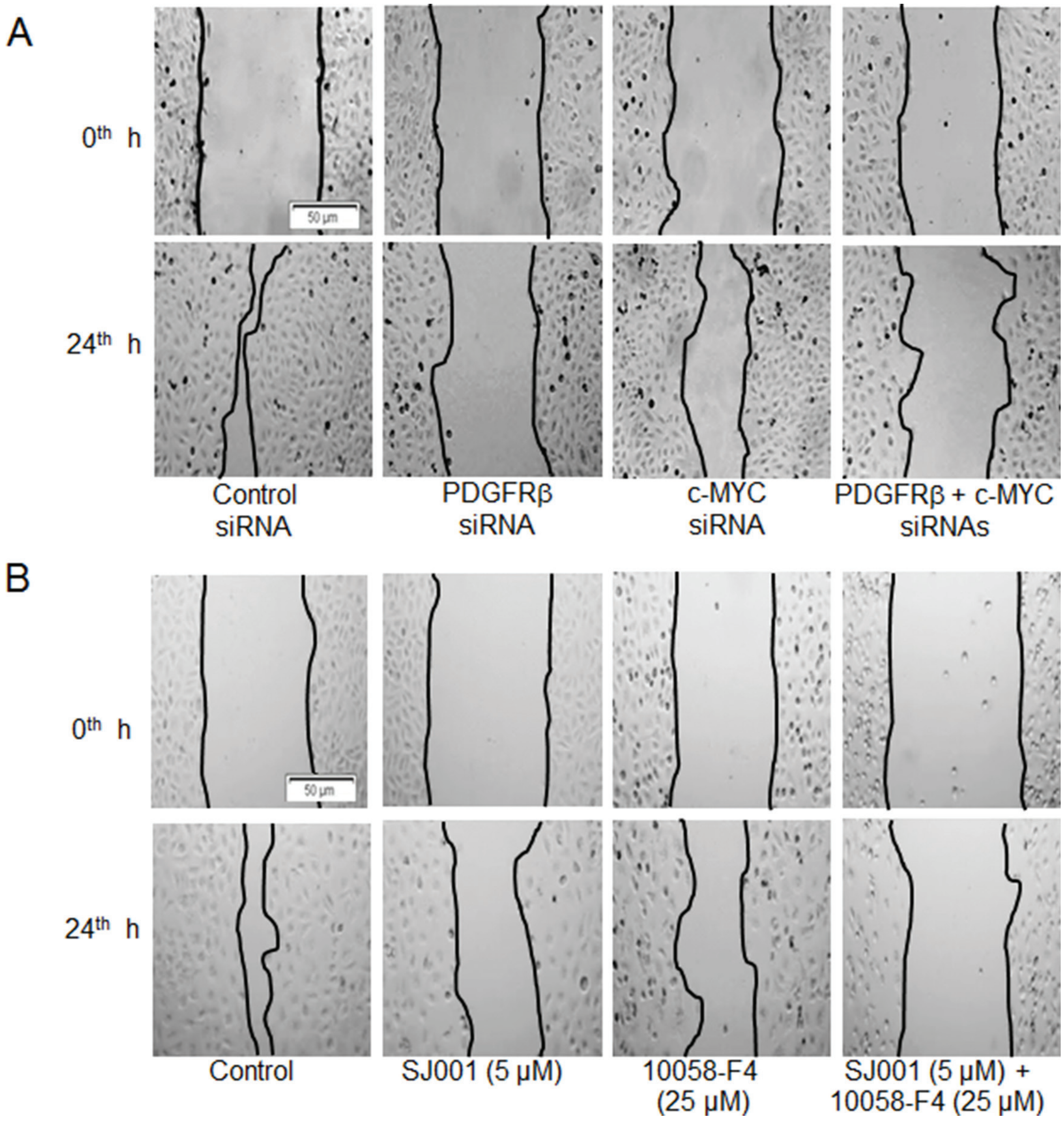

Supplementary Figure S3: The representative figures of co-targeting PDGFR and c-MYC on MB cell migration. Daoy cells were treated with siRNAs for PDGFRβ and c-MYC and the specific inhibitors of PDGFRβ and c-MYC for 36 h. Treated cells were then detached and re-distributed in equal amounts in a 48-well plate before a linear wound was made. The images were captured immediately after that an artificial wound was made at 0th h and also at 24th h. The experiments were repeated for 3 times. The data are representative images for each condition.

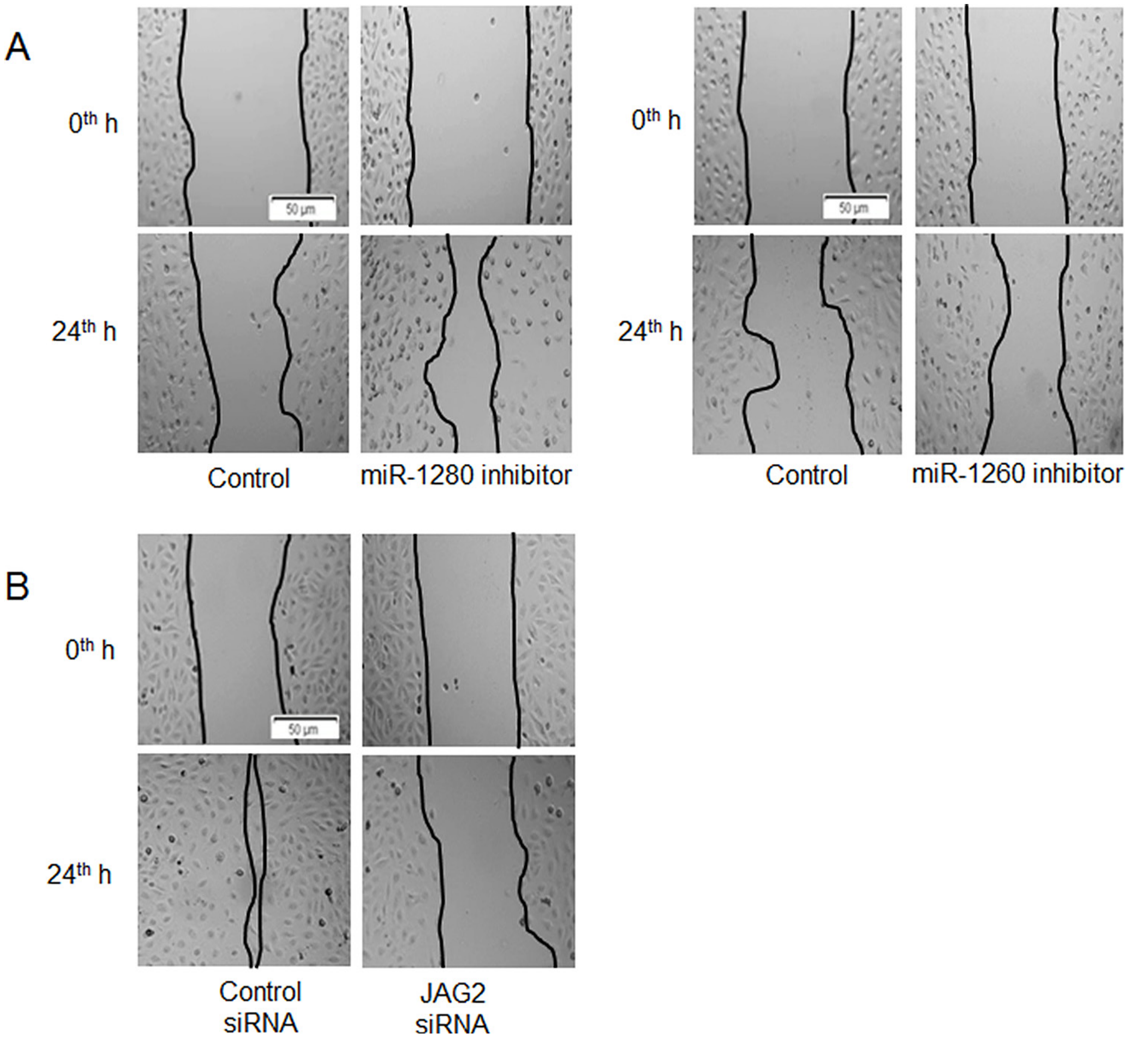

Supplementary Figure S4: The representative figures of miR-1280 inhibitors and JAG2 siRNA on MB cell migration. PDGFRβ^{KD} Daoy cells were treated with either inhibitor of miR-1280, or JAG2 siRNA for 36 h and then detached and re-distributed in equal amounts in a 48-well plate before a linear wound was made. The images were captured immediately after that an artificial wound was made at 0th h and also at 24th h. The experiments were repeated for 3 times. The data are representative images for each condition.

Supplementary Table S1: The *p* values of subgroup comparisons for the Heidelberg dataset in Figure 1

PDGFRα – Boston cohort

Comparison	Posthoc test (*p*-value)
NCB vs WNT	0.06
NCB vs SHH	**0.01**
NCB vs Group 3	**1.60e-09**
NCB vs Group 4	**3.70e-04**
WNT vs SHH	0.83
WNT vs Group 3	**1.70e-12**
WNT vs Group 4	**1.20e-08**
SHH vs Group 3	**1.90e-24**
SHH vs Group 4	**8.80e-19**
Group 3 vs Group 4	**0.02**

PDGFRβ – Boston cohort

Comparison	Posthoc test (*p*-value)
NCB vs WNT	0.10
NCB vs SHH	**1.4e-03**
NCB vs Group 3	0.13
NCB vs Group 4	0.24
WNT vs SHH	0.17
WNT vs Group 3	0.70
WNT vs Group 4	0.41
SHH vs Group 3	**8.2e-03**
SHH vs Group 4	**4.3e-04**
Group 3 vs Group 4	0.51

PDGFRα – Heidelberg cohort

Comparison	Posthoc test (*p*-value)
WNT vs SHH	0.85
WNT vs Group 3	**3.7e-03**
WNT vs Group 4	**7.2e-04**
SHH vs Group 3	**1.6e-03**
SHH vs Group 4	**1.9e-04**
Group 3 vs Group 4	0.33

PDGFRβ – Heidelberg cohort

Comparison	Posthoc test (*p*-value)
WNT vs SHH	**2.6e-06**
WNT vs Group 3	0.48
WNT vs Group 4	0.31
SHH vs Group 3	**6.5e-04**
SHH vs Group 4	**2.6e-05**
Group 3 vs Group 4	0.98

Supplementary Table S2: Pathway analysis of genes co-expressed with PDGFRα in MB tumors (*indicates genes in the pathways that are specific for PDGFRα.)

Supplementary Table S3: Pathway analysis of genes co-expressed with PDGFRβ in MB tumors (*indicates genes in the pathways that are specific for PDGFRβ.)

Supplementary Table S4: Pathway analysis of genes co-expressed with c-MYC in MB tumors
